# 

**Published:** 1951-07

**Authors:** 


### CONTENTS

| | Page |
|---|---|
| **The Atomic Bomb.** I.—THE RELEASE OF ATOMIC ENERGY AND THE PATHOLOGICAL EFFECTS OF RADIATION: By R. C. TUDWAY, M.B., B.S., B.Sc., D.M.R. II.—THE EFFECTS OF THE EXPLOSION: By JOHN H. CHALLENGER, M.B., Ch.B., D.A. | 71 |
| **Reiter's Syndrome.** By A. E. W. McLACHLAN, M.D., D.P.H., F.R.S.E. | 87 |
| **Backache, Sciatica and Ruptured Discs.** By KENNETH PRIDIE, F.R.C.S. | 95 |
| **Editorial** | 98 |
| **Meetings of Societies** | 100 |
| **Local Medical Notes** | 102 |

